# Inhalation of the Rho-kinase inhibitor Y-27632 reverses allergen-induced airway hyperresponsiveness after the early and late asthmatic reaction

**DOI:** 10.1186/1465-9921-7-121

**Published:** 2006-09-26

**Authors:** Dedmer Schaafsma, I Sophie T Bos, Annet B Zuidhof, Johan Zaagsma, Herman Meurs

**Affiliations:** 1 Department of Molecular Pharmacology, University of Groningen, Antonius Deusinglaan 1, 9713 AV Groningen, The Netherlands

## Abstract

**Background:**

In guinea pigs, we have previously demonstrated that the contribution of Rho-kinase to airway responsiveness *in vivo *and *ex vivo *is enhanced after active sensitization with ovalbumin (OA). Using conscious, unrestrained OA-sensitized guina pigs, we now investigated the role of Rho-kinase in the development of airway hyperresponsiveness (AHR) after the allergen-induced early (EAR) and late asthmatic reaction (LAR) *in vivo*.

**Methods:**

Histamine and PGF_2α _PC_100_-values (provocation concentrations causing 100% increase in pleural pressure) were assessed before OA-challenge (basal airway responsiveness) and after the OA-induced EAR (5 h after challenge) and LAR (23 h after challenge). Thirty minutes later, saline or the specific Rho-kinase inhibitor Y-27632 (5 mM, nebulizer concentration) were nebulized, after which PC_100_-values were reassessed.

**Results:**

In contrast to saline, Y-27632 inhalation significantly decreased the basal responsiveness toward histamine and PGF_2α _before OA-challenge, as indicated by increased PC_100 _-values. Both after the allergen-induced EAR and LAR, AHR to histamine and PGF_2α _was present, which was reversed by Y-27632 inhalation. Moreover, there was an increased effectiveness of Y-27632 to reduce airway responsiveness to histamine and PGF_2α _after the EAR and LAR as compared to pre-challenge conditions. Saline inhalations did not affect histamine or PGF_2α _PC_100_-values at all. Interestingly, under all conditions Y-27632 was significantly more effective in reducing airway responsiveness to PGF_2α _as compared to histamine. Also, there was a clear tendency (P = 0.08) to a more pronounced degree of AHR after the EAR for PGF_2α _than for histamine.

**Conclusion:**

The results indicate that inhalation of the Rho-kinase inhibitor Y-27632 causes a considerable bronchoprotection to both histamine and PGF_2α_. Moreover, the results are indicative of a differential involvement of Rho-kinase in the agonist-induced airway obstruction *in vivo*. Increased Rho-kinase activity contributes to the allergen-induced AHR to histamine and PGF_2α _after both the EAR and the LAR, which is effectively reversed by inhalation of Y-27632. Therefore, Rho-kinase can be considered as a potential pharmacotherapeutical target in allergic asthma.

## Background

Asthma is an inflammatory airways disease characterized by airway hyperresponsiveness (AHR) to a variety of stimuli, including contractile agonists such as histamine and prostaglandin F_2α _(PGF_2α_) [1-4].

Agonist-induced smooth muscle contraction is largely governed by phosphorylation of the 20kDa myosin light chain (MLC_20_) [5]. MLC_20 _phosphorylation is initiated by an increase in intracellular Ca^2+^-concentration ([Ca^2+^]_i_) and subsequent formation of Ca^2+^-calmodulin, resulting in activation of myosin light chain kinase (MLCK). The extent of MLC_20 _phosphorylation is determined by the balance between MLCK and myosin light chain phosphatase (MLCP) activities [6]. Recently, it has been established that contractile stimuli do not exert their effects only by increasing [Ca^2+^]_i_, but also by increasing the sensitivity of the contractile apparatus to Ca^2+^. One of the main pathways involved in this Ca^2+^-sensitization is the RhoA/Rho-kinase pathway [7,6]. Activated Rho-kinase interferes with the equilibrium of MLCK and MLCP activities by phosphorylating and thereby inactivating the myosin binding subunit of MLCP. This leads to an augmentation of MLC_20 _phosphorylation and hence an elevated level of contraction at an established [Ca^2+^]_i _[7,8].

*In vitro *studies have indicated a receptor-dependent role of Rho-kinase in agonist-induced airway smooth muscle (ASM) contraction. Thus, the potency and maximal effect of histamine-induced contraction of guinea pig tracheal smooth muscle preparations were unaffected by inhibition of Rho-kinase, whereas these parameters were strongly dependent on Rho-kinase for PGF_2α_-induced contraction [9]. Growth factor-induced contraction of human and guinea pig ASM preparations appeared to be almost completely dependent on Rho-kinase [10,11]. presumably via generation of contractile prostaglandins [11]. Thusfar, no reports have been published on a differential role for Rho-kinase in airway responsiveneness to contractile agonists *in vivo*.

Recently, Rho-kinase has emerged to be a potential target in airways diseases, including allergic asthma [12]. *Ex vivo*, it has been demonstrated that Rho/Rho-kinase-mediated Ca^2+^-sensitization is enhanced in acetylcholine-induced contraction of bronchial smooth muscle obtained from repeatedly allergen-challenged rats [13]. Moreover, we have recently demonstrated that active allergic sensitization (without subsequent allergen exposure) increased contractile potency of guinea pig tracheal smooth muscle preparations towards histamine and PGF_2α _in a Rho-kinase dependent fashion. Similarly, passive sensitization-induced nonspecific ASM hyperresponsiveness and specific allergen responsiveness in these preparations were found to be dependent on Rho-kinase as well [14]. Also *in vivo*, using permanently instrumented, unanaesthetized, unrestrained guinea pigs, we found that the contribution of Rho-kinase to airway responsiveness to histamine was augmented after active allergic sensitization [9]. However, the contribution of Rho-kinase to the development of AHR after the allergen-induced early (EAR) and late (LAR) asthmatic reaction in this model is presently unknown.

In the present study, using the same model, we therefore investigated the involvement of Rho-kinase in the airway responsiveneness to histamine and PGF_2α _before and after the allergen-induced EAR and LAR. We demonstrate that there is a differential role of Rho-kinase in the agonist-induced airway obstructions and that inhalation of the specific Rho-kinase inhibitorY-27632 results in a strong bronchoprotection to both agonists Moreover, the results indicate that increased Rho-kinase activity contributes to allergen-induced AHR to histamine and PGF_2α _after both the EAR and the LAR, which is effectively reversed by Y-27632 inhalation.

## Methods

### Animals

Outbred specified pathogen-free male Dunkin Hartley guinea pigs (Harlan, Heathfield, U.K.), weighing 500–700 g, were used in this study. The animals were actively IgE-sensitized to ovalbumin (OA) as described previously [15]. In short, 0.5 ml of an allergen solution containing 100 μg/ml OA and 100 mg/ml Al(OH)_3 _in saline was injected intraperitoneally, while another 0.5 ml was divided over seven intracutaneous injection sites in the proximity of lymph nodes in the paws, lumbar regions and the neck. The animals were operated 2 weeks after sensitization and used experimentally in weeks 4 to 8 after sensitization. The animals were group-housed in individual cages in climate controlled animal quarters and given water and food *ad libitum*, while a 12-h on/12-h off light cycle was maintained. All protocols described in this study were approved by the University of Groningen Committee for Animal Experimentation.

### Measurement of airway function

Airway function was assessed in conscious, permanently instrumented, unrestrained guinea pigs, by on-line measurement of pleural pressure (P_pl_) as described previously [16]. In short, a small saline-filled balloon-catheter was surgically implanted inside the thoracic cavity. The free end of the catheter was driven subcutaneously to the neck of the animal, where it was exposed and attached permanently. Via an external saline-filled canula the pleural balloon was connected to a pressure transducer (Ohmeda DTX, SpectraMed, Bilthoven, the Netherlands) and an on-line computer system, enabling continuous measurement of P_pl _changes (in cm H_2_O). We have previously found that changes in P_pl _are linearly correlated with changes in airway resistance and hence can be used as a sensitive index for bronchoconstriction [16].

### Provocation procedures

Provocations with OA, histamine and PGF_2α_, as well as administration of Y-27632 were performed by inhalation of aerosolized solutions. Aerosols were produced by a DeVilbiss nebulizer (type 646; DeVilbiss, Somerset, PA, USA), driven by an airflow of 8 l/min and resulting in an output of 0.33 ml/min. Provocations were carried out in a perspex cage (internal volume of 9 l) in which the guinea pigs could move freely [16]. Before the start of the experiment, the animals were habituated to the experimental conditions on two sequential days at least one week after surgery, when preoperative weight had been restored. On the first day, the animals were placed in the provocation cage unconnected to the pressure transducer. After an adaptation period of at least 30 min, three consecutive provocations with saline were performed, each exposure lasting 3 min and separated by a 7-min interval. The next day, this procedure was repeated with the animals connected to the measurement system.

On the experimental days, following the habituation procedure, OA, histamine and PGF_2α _provocations were performed as described below. All provocations were preceded by an adaptation period of at least 30 min, followed by two consecutive control provocations with saline as described above. Baseline Ppl was calculated by averaging the Ppl of the last 20 min of the adaptation period.

To assess the airway reactivity to histamine, provocations were performed with an initial 25 μg/ml histamine solution in saline, followed by increasing dosage steps of 25 μg/ml. Histamine provocations lasted 3 min, separated by 7 min intervals. Animals were challenged until P_pl _was increased by more than 100 % above baseline for at least 3 consecutive minutes. P_pl _returned to baseline value within 15 min after the last provocation. The provocation concentration causing a 100 % increase of P_pl _(PC_100_-value) was derived by linear intrapolation of the concentration-P_pl _curve and was used as a measure for airway reactivity toward the agonist. Using the same procedure, airway reactivity to PGF_2α _was determined by using increasing concentrations of 1.25, 2.5, 5, 10, 15, 20, 37.5, 50, 75, 100 and 125 μg/ml of PGF_2α _in saline, respectively. OA-provocations were performed by inhalation of increasing aerosol concentrations of 0.5 and 1.0 mg/ml OA in saline for 3 min, separated by 7 min intervals. Allergen inhalations were discontinued when an increase in P_pl _of more than 100 % was observed. Using these conditions, none of the animals developed anaphylactic shock after allergen provocation.

### Provocation protocol

On two different occasions, separated by a one week interval, histamine or PGF_2α _PC_100_-values were assessed 24 h before OA-challenge, and at 5 h and 23 h after the OA-challenge, *i.e. *after the early (EAR) and late (LAR) asthmatic reaction, respectively. Thirty minutes after each histamine or PGF_2α _inhalation, saline or Y-27632 (5 mM) was nebulized during 3 min, followed by reassessment of the histamine or PGF_2α _PC_100_-values 30 min later. Saline and Y-27632 inhalations were alternated using a random crossover design.

### Data analysis

All data represent means ± s.e. mean from *n *separate experiments. Statistical significance of differences was evaluated using a repeated measures one way analysis of variance (ANOVA) followed by a Holm-Sidak post-test, and significance was accepted when *P *< 0.05.

### Chemicals

Ovalbumin (grade III) and histamine dihydrochloride were obtained from Sigma Chemical Co. (St. Louis, MO, U.S.A.). PGF_2α _was obtained from Pharmacia and Upjohn (Puurs, Belgium) and (+)-(R)-trans-4-(1-aminoethyl)-N-(4-pyridyl) cyclohexane carboxamide (Y-27632) was obtained from Tocris Cookson Ltd. (Bristol, U.K.). All other chemicals were of analytical grade.

## Results

**Figure 1:**
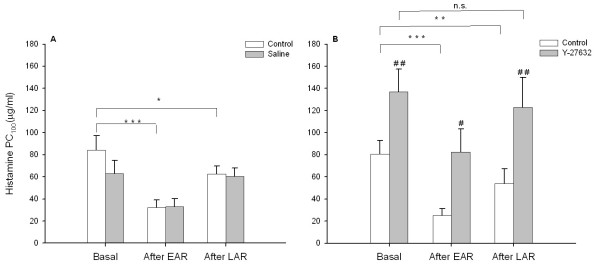
Effects of saline (A) and Y-27632 (5 mM nebulizer concentration; B) inhalations on airway responsiveness toward histamine after the allergen-induced EAR and LAR. Data represent means ± s.e.mean of 5 animals. *P < 0.05, **P < 0.01, ***P < 0.001 compared to basal; ^#^P < 0.05, ^## ^P < 0.01 compared to control.

In contrast to saline (Fig. 1A), Y-27632 significantly decreased the basal responsiveness toward histamine before OA-challenge, as indicated by an increased PC_100 _(Fig. 1B). After the EAR, AHR had developed (Fig. 1A and 1B), which was reversed by Y-27632 to the level of basal responsiveness in the absence of the Rho-kinase inhibitor (Fig. 1B). Interestingly, the AHR after the LAR was even fully reversed to the basal responsiveness in the presence of Y-27632 (Fig. 1B). Saline inhalations did not affect histamine PC_100_-values after the EAR and LAR (Fig. 1A).

**Figure 2:**
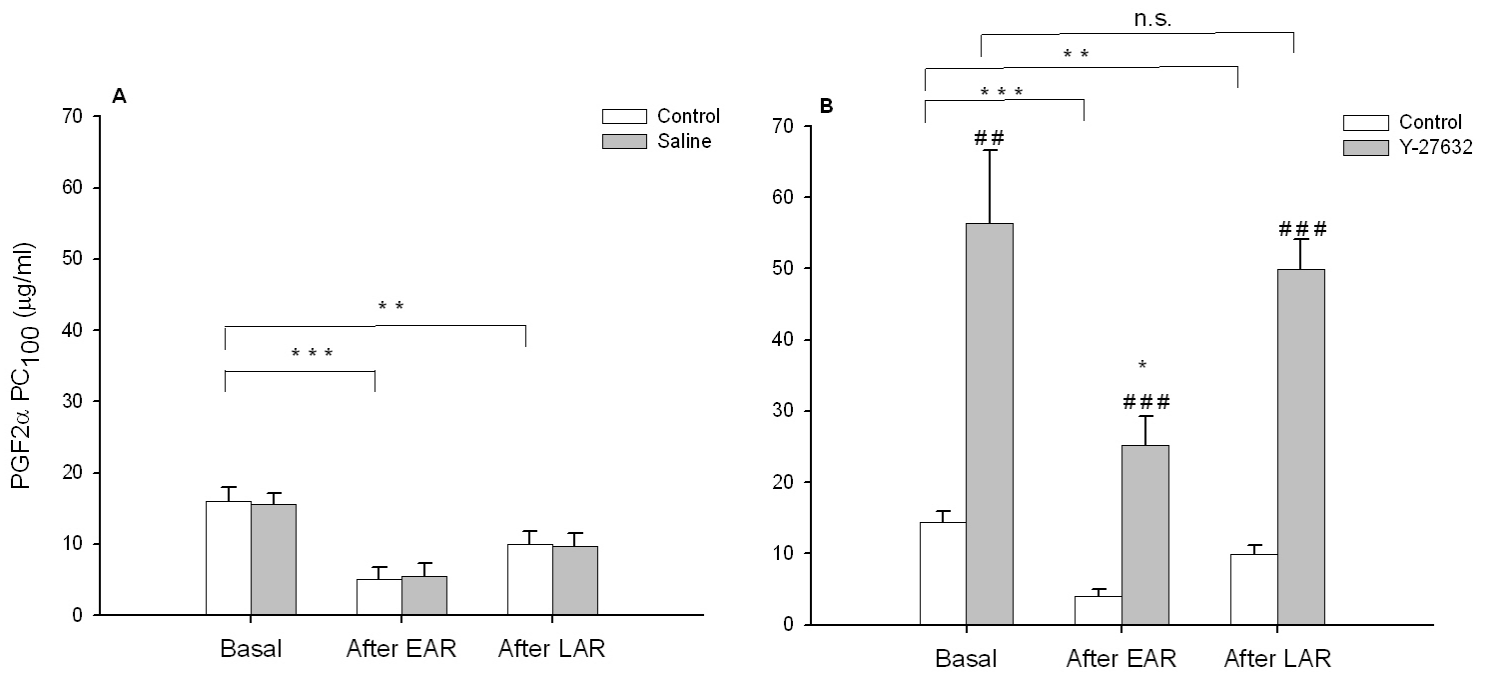
Effects of saline (A) and Y-27632 (5 mM nebulizer concentration; B) inhalations on airway responsiveness toward PGF_2α _after the allergen-induced EAR and LAR. Data represent means ± s.e.mean of 7 animals. *P < 0.05, **P < 0.01, ***P < 0.001 compared to basal; ^##^P < 0.01, ^### ^P < 0.001 compared to control.

As shown in figure 2B, basal responsiveness to PGF_2α _was also significantly inhibited by Y-27632 inhalation to a considerable extent. The AHR after the EAR was strongly reversed by Y-27632 inhalation to a hyporesponsive level as compared to basal airway responsiveness in the absence of the Rho-kinase inhibitor (P < 0.05). As for histamine, Y-27632 inhalation fully reversed the AHR to PGF_2α _after the LAR to the basal responsiveness as measured in the presence of Y-27632 (Fig. 2B). As with histamine, saline inhalations did not affect PC_100 _values for PGF_2α _(Fig. 2A).

**Figure 3:**
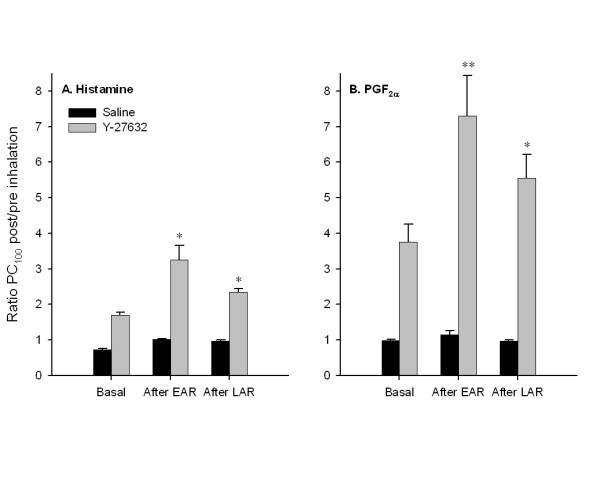
Effectiveness of saline and Y-27632 inhalations to reduce basal airway responsiveness and airway (hyper)responsiveness after the EAR and the LAR for histamine (A) and PGF_2α _(B). Data are expressed as the PC_100 _ratio post/pre saline or Y-27632 inhalation. Data represent means ± s.e.mean of 5 (histamine) and 7 (PGF_2α_) animals. *P < 0.05 **P < 0.01 compared to basal effectiveness.

As compared to basal conditions (1.7 ± 0.1-fold decrease of airway responsiveness), the effectiveness of Y-27632 to reduce the airway responsiveness to histamine after the EAR (3.3 ± 0.4-fold) and after the LAR (2.3 ± 0.1-fold) was significantly increased (P < 0.05 both; Fig. 3A). Also for PGF_2α_, Y-27632 inhalation was much more effective in reducing the airway responsiveness after the EAR (7.3 ± 1.1-fold, P < 0.05) and the LAR (5.6 ± 0.7-fold, P < 0.05) as compared to pre-challenge conditions (3.8 ± 0.5-fold decrease, Fig. 3B). Interestingly, under all conditions Y-27632 was significantly more effective in reducing airway responsiveness to PGF_2α _as compared to histamine, indicating that there is a receptor-dependent role for Rho-kinase in airway responsiveness *in vivo*. In addition, we found that there is a clear tendency (P = 0.08) for a more pronounced degree of AHR after the EAR for PGF_2α _(5.8 ± 1.1-fold increase in airway reactivity) than for histamine (3.3 ± 0.4-fold increase in airway reactivity). No difference was observed in the degree of AHR after the LAR for both agonists (1.7 ± 0.2 and 1.6 ± 0.2 for PGF_2α _and histamine, respectively).

## Discussion

In the present study, we demonstrated that inhalation of the Rho-kinase inhibitor Y-27632 causes a considerable bronchoprotection against histamine and PGF_2α _under basal conditions. Moreover, we showed for the first time that in conscious, freely moving, actively OA-sensitized guinea pigs, inhalation of the Rho-kinase inhibitor Y-27632 reverses the AHR to both agonists after the allergen-induced EAR and LAR. The results strongly indicate that an increased Rho-kinase activity is involved in the development of the allergen challenge-induced AHR, as demonstrated by an enhanced effectiveness of Y-27632 to inhibit the increased airway responsiveness to histamine and PGF_2α_, both after the EAR and the LAR.

Further investigations are warranted to reveal the exact mechanisms underlying the increased contribution of Rho-kinase to airway responsiveness after the EAR and LAR. Evidence exists that allergic sensitization by itself is already a key process in augmenting the role of Rho-kinase in contractile airway responsiveness. Thus, we previously found that active allergic sensitization by itself, without subsequent allergen exposure, is sufficient to induce an enhanced role of Rho-kinase in guinea pig airway smooth muscle contraction *ex vivo *and airway responsiveness *in vivo *[9]. Also in passively sensitized guinea pig tracheal preparations, we recently found that the nonspecific hyperresponsiveness in response to histamine and methacholine was fully normalized by Rho-kinase inhibition [14]. The enhanced contribution of Rho-kinase to airway responsiveness could involve increased expression of RhoA, as protein levels of this upstream activator of Rho-kinase have been reported elevated both after allergic sensitization in guinea pigs [9] and after repeated allergen challenge in rats [13] and mice [17]. Inflammatory cells – activated during the allergic reaction – release mediators, including prostaglandins, leukotrienes and growth factors [18,19], which have been reported to be dependent on Rho-kinase for their contractile effects [10,11]. It can be envisaged that there is synergism in Rho-kinase activation between such mediators and the inhaled agonists, which results in a higher efficacy of Rho-kinase inhibition. In addition, Rho-kinase inhibition might have effects on airway inflammation itself, as has been suggested in a murine model of acute allergic airway inflammation. In anaesthetized mice, it was found that when Y-27632 was given intranasally *prior *to allergen challenge, pulmonary eosinophilia was reduced, as shown by a decreased number of eosinophils in the bronchoalveolar lavage (BAL) fluid [20]. In the same study, it was also demonstrated that intranasally administered Y-27632, which was given before every allergen challenge, reduced the repeated allergen-induced increased responsiveness to intravenously applied methacholine, which might be correlated to effects on airways inflammation [20]. Also, it has been demonstrated *in vitro *that Y-27632 decreased the release of the Th2 cytokines IL-4 and IL-5 [21].

It has been previously reported that a differential contribution of Rho-kinase to histamine- and PGF_2α_-induced ASM contraction exists *in vitro *[9]. Fully in line with those findings, we found that such a differential role of Rho-kinase also exists *in vivo*. Thus, under all conditions inhalation of Y-27632 was significantly more effective in reducing airway responsiveness to PGF_2α _as compared to histamine. Moreover, there was a strong tendency to a more pronounced AHR after the EAR in response to PGF_2α _as compared to histamine. Together with the higher efficacy by which Y-27632 inhalation reduces airway responsiveness to PGF_2α _as compared to histamine, this might suggest that the severity of AHR to a certain agonist is associated with the extent to which the agonist is dependent on Rho-kinase for its contractile effect.

## Conclusion

Inhalation of the Rho-kinase inhibitor Y-27632 causes a considerable bronchoprotection to histamine and PGF_2α_. Moreover, a differential involvement of Rho-kinase in the contractile agonist-induced airway obstructions exists *in vivo*. Increased Rho-kinase activity contributes to the allergen-induced AHR to histamine and PGF_2α _after both the EAR and the LAR, which is effectively reversed by inhalation of Y-27632. Therefore, Rho-kinase can be considered as a potential pharmacotherapeutical target in allergic asthma.

## Abbreviations

AHR, airway hyperresponsiveness; ASM, airway smooth muscle; EAR, early asthmatic reaction; LAR, late asthmatic reaction; MLC, myosin light chain; PC_100_, provocation concentration causing 100 % increase in pleural pressure; P_pl_, pleural pressure; PGF_2α_, prostaglandin F_2α_;KH, Krebs-Henseleit; OA, ovalbumin

## Competing interests

The author(s) declare that they have no competing interests.

## Authors' contributions

DS designed and coordinated the study, performed a major part of the experiments, performed the statistical analysis and drafted the manuscript. ISTB and ABZ substantially assisted in performing the experiments. JZ participated in the design of the study and the interpretation of results. HM supervised the study, participated in its design and in interpretation of results as well as in the preparation of the manuscript. All authors read and approved the final manuscript.
